# Supplementary material for: Causal Relationship between Aging and Anorexia Nervosa: A White-Matter-Microstructure-Mediated Mendelian Randomization Analysis
Source: Biomedicines. 2024 Aug 16;12(8):1874. doi: 10.3390/biomedicines12081874 (PMC11351342; doi:10.3390/biomedicines12081874)
Supplement: Supplementary file 1 [file biomedicines-12-01874-s001.zip › Supplementary Figure/Figure S8/Leaveoneout plot of MR analysis for TL to ubm-b-1932.pdf]

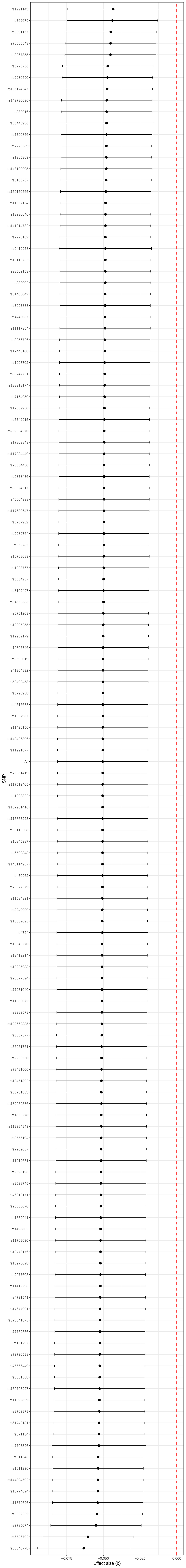

rs1291143
rs762679
rs3891167
rs76065543
rs2967355
rs6776756
rs2230590
rs185174247
rs142730696
rs939916
rs35446936
rs7790856
rs7772289
rs1985369
rs143190905
rs8105767
rs150150565
rs11557154
rs13230646
rs141214782
rs2276182
rs9419958
rs10112752
rs28502153
rs932002
rs61405042
rs3093888
rs4743037
rs11117354
rs2056726
rs17445108
rs1907702
rs55747751
rs188918174
rs7164950
rs12369950
rs5742915
rs202034370
rs17803849
rs117034449
rs75664430
rs9878436
rs80324517
rs45604339
rs117630647
rs3767952
rs2282764
rs869785
rs10768683
rs1023767
rs6054257
rs8102497
rs34550383
rs6751209
rs10905255
rs12932179
rs10805346
rs9600019
rs41304832
rs59409453
rs6790988
rs4616688
rs1957937
rs11426156
rs142426306
rs11991877
All
rs73581419
rs117512405
rs1003322
rs137901416
rs116863223
rs80116508
rs10845387
rs6590343
rs145114957
rs450962
rs79977579
rs11584821
rs9940099
rs13062095
rs4724
rs10840270
rs12412214
rs12925933
rs28577594
rs77231040
rs11085072
rs2293579
rs139669835
rs6587577
rs56061761
rs9955360
rs78491606
rs12451892
rs66731853
rs182059586
rs4530278
rs112394943
rs2555104
rs7209057
rs11212631
rs9398196
rs2538745
rs76219171
rs28363070
rs1332941
rs4498805
rs11769630
rs10773176
rs16978028
rs2977608
rs11412296
rs4731541
rs17677991
rs376641875
rs77732866
rs131797
rs73730598
rs76666449
rs6881568
rs139795227
rs11699829
rs2763979
rs61748181
rs871134
rs7705526
rs611646
rs1611236
rs144204502
rs10774624
rs11579626
rs6669563
rs3785074
rs6536702
rs35640778
SNP
−0.075
−0.050
−0.025
0.000
Effect size (b)
